# Clinical Characteristics of Adolescents Admitted to a Child and Adolescent Psychiatry Department in Poland: A Retrospective Chart Review

**DOI:** 10.3390/jcm15093493

**Published:** 2026-05-02

**Authors:** Magdalena Uzar, Weronika Zwolińska, Tomasz Hałas, Aleksandra Hajdo-Kołbuc, Agnieszka Słopień

**Affiliations:** 1 Department of Child and Adolescent Psychiatry, Karol Jonscher Clinical Hospital, Poznan University of Medical Sciences, 27/33 Szpitalna St., 60-572 Poznan, Poland; wzwolinska@ump.edu.pl (W.Z.); halastomek@wp.pl (T.H.); aleksandra.hajdo@gmail.com (A.H.-K.); agaslopien@ump.edu.pl (A.S.); 2 Doctoral School, Poznan University of Medical Sciences, 70 Bukowska St., 60-812 Poznan, Poland

**Keywords:** emotional dysregulation, adolescents, emergency psychiatry

## Abstract

**Background/Objectives:** Adolescents admitted for emergency psychiatric hospitalization frequently present with severe and heterogeneous psychopathology. In clinical practice, some adolescent inpatients appear to present a broader symptom pattern suggestive of emotional dysregulation. However, it remains unclear whether they can truly be distinguished in this population and whether they differ meaningfully from adolescents with predominantly depressive presentations. **Methods:** We conducted a retrospective cross-sectional chart review with subgroup analysis based on the medical records of patients aged 11–17 years hospitalized on an emergency basis at the Department of Child and Adolescent Psychiatry in Poznań, Poland, between January and December 2024. Patients were assigned either to an emotional dysregulation group, defined by affective dysregulation and behavioral dyscontrol, or to a depressive presentations group, comprising adolescents with depressive presentations who did not meet criteria for the emotional dysregulation profile. Broader clinical characteristics, adverse childhood experiences, and prior treatment history were compared between groups. **Results:** A total of 139 adolescents were included (85 in the emotional dysregulation group and 54 in the depressive presentations group). The median age was 13 years [Q1–Q3: 13–14] in the emotional dysregulation group and 14 years [Q1–Q3: 12.25–14] in the depressive presentations group; girls comprised 77.6% and 83.3% of the groups, respectively. The emotional dysregulation group more often presented with conflict-ridden relationships, a more frequent history of suicide attempts (72.9% vs. 50.0%, *p* = 0.006), and a higher number of suicide attempts (median 1 [Q1–Q3: 0–2] vs. 0.5 [Q1–Q3: 0–1], *p* = 0.012), as well as more frequent exposure to adversity-related experiences. Furthermore, this group had a higher number of previous psychiatric hospitalizations (median 1 [Q1–Q3: 1–2] vs. 1 [Q1–Q3: 1–1], *p* = 0.001) and a longer history of psychiatric treatment. In contrast, social withdrawal was more characteristic of the depressive presentations group. **Conclusions:** Routinely collected clinical records may capture a clinically meaningful subgroup of adolescents with a symptom profile suggestive of emotional dysregulation. Compared with the depressive presentations group, these adolescents showed greater interpersonal difficulties, more recurrent suicide attempts, greater adversity burden, and a longer history of psychiatric treatment. Further prospective studies using standardized measures are needed.

## 1. Introduction

In Poland, one pathway to child and adolescent psychiatric inpatient care is via emergency hospitalization, which is defined as an admission due to severe psychiatric symptoms, typically when the patient’s condition poses a risk to their own health or life or to the safety of others [1,2]. Research indicates that emergency admissions account for a substantial proportion of psychiatric hospitalizations among young patients in Poland and that their number has been increasing in recent years. According to reports by the Ministry of Health, the percentage of emergency admissions of children and adolescents to psychiatric wards in 2016 was 64.1%, whereas by 2024, it had increased to 74.46% [3,4]. Similar trends have also been observed in other countries. International data, however, remain heterogeneous and not directly comparable across healthcare systems, having been derived from non-equivalent service settings. Such data include emergency department visits, psychiatric emergency admissions, and acute medical ward admissions for mental health concerns. Reported increases include an 89.1% rise in mental health-related emergency department visits among children and adolescents in Ontario, Canada, between 2006 and 2017 [5], and a 28% increase in such visits in the United States between 2011 and 2015 [6]. In addition, emergency admissions increased by 405% in the Child and Adolescent Psychiatry Clinic in Tübingen between 1996 and 2014 [7], while admissions to acute medical units in England for mental health concerns rose by 65.0% between 2012/13 and 2021/22 [8].

Previous studies have shown that children and adolescents admitted for emergency psychiatric care often present with severe psychopathology, including marked emotional reactivity, self-harming behaviors, and suicidal thoughts and behaviors, as well as aggression [9]. Some authors have suggested that difficulties in emotion regulation may be particularly prominent in a subgroup of adolescents requiring acute psychiatric care [10].

Emotion regulation refers to the capability to recognize and accept one’s emotional states, the capacity to control impulsive behavior, the ability to act in accordance with one’s goals despite emotional distress, and the flexibility to use regulatory strategies in accordance with the given circumstances [11]. Emotional dysregulation, on the other hand, refers to a pattern of difficulties in monitoring, evaluating, and modulating emotional experiences and responses in ways that support adaptive functioning and the attainment of personally relevant goals [11,12]. In children and adolescents, such difficulties may manifest as affective instability, irritability, heightened emotional reactivity, and poor behavioral control. Additionally, they may be associated with aggression, self-harm, and interpersonal problems [13,14,15,16]. Emotional dysregulation is increasingly conceptualized as a transdiagnostic phenomenon that has been observed across a wide variety of mental disorders, including mood disorders, borderline personality disorder, eating disorders, and ADHD [17,18,19]. Some evidence also suggests that emotion regulation difficulties may be highly prevalent among psychiatric inpatients [20].

From a clinical perspective, emotional dysregulation may therefore represent an important clinical dimension in acutely hospitalized adolescents, particularly in patients whose presentations do not clearly fit within a single diagnostic category. This issue may be particularly relevant in routine psychiatric practice, where adolescents with marked affective instability, impulsivity, self-harm, aggression, and interpersonal difficulties often receive multiple, changing, or nonspecific diagnoses.

Adverse childhood experiences (ACEs) traditionally refer to abuse, neglect, and household dysfunction occurring before the age of 18 [21]. Broader formulations of childhood adversity may additionally include adverse peer experiences, accidents, and exposure to terrorism and war [22]. A large body of research indicates that ACEs may negatively impact the development and behavior of children and adolescents and are significantly associated with a wide range of mental and somatic health outcomes [23,24,25,26]. Growing evidence also points to a close association between childhood adversity and emotional dysregulation. Keeshin et al. highlighted that individuals who experienced childhood trauma are more likely to develop emotion regulation difficulties, while a substantial proportion of patients with emotional dysregulation report a history of ACEs [27].

In everyday clinical practice, some adolescents hospitalized in psychiatric wards appear to present a broader symptom pattern characterized by affective instability, irritability, impulsivity, aggressive and self-harming behaviors, interpersonal difficulties, and, sometimes, transient dissociative and brief stress-related psychotic-like symptoms. Although such patients also frequently report depressive and anxiety symptoms, their overall presentation may be broader and more complex than that typically observed in adolescents with predominantly depressive symptomatology.

At the time of data collection, ICD-10 remained the diagnostic framework used in routine clinical documentation in Poland. Within this framework, such adolescents may receive a range of diagnoses, including mixed disorders of conduct and emotions, unspecified emotional disorders of childhood, unspecified personality disorder, attachment disorders, depressive disorders, mixed anxiety and depressive disorder, post-traumatic stress disorder, or adjustment disorders. Despite this diagnostic heterogeneity, some of them may share a clinically meaningful profile characterized by emotional dysregulation, interpersonal difficulties, and a broader functional impairment. In the present study, this profile was not conceptualized as a formal diagnostic category but rather as a transdiagnostic, symptom-based clinical pattern identifiable across heterogeneous ICD-10 diagnoses and extending beyond depressive symptomatology alone.

Previous studies suggest that emotional dysregulation, self-harm, suicidality, childhood adversity, and externalizing symptoms are common among adolescents requiring acute psychiatric care. Nonetheless, it remains unclear whether a clinically meaningful subgroup characterized by prominent emotional dysregulation can be identified among adolescents hospitalized in emergency psychiatric settings. Moreover, it has not been determined whether such patients differ from adolescents with predominantly depressive presentations in terms of broader clinical characteristics, exposure to ACEs, and prior treatment history.

The aim of the present study was to examine whether adolescents with a retrospectively identified symptom profile suggestive of emotional dysregulation differ from adolescents with predominantly depressive presentations, but without the broader dysregulation profile, with regard to clinical characteristics, adverse childhood experiences, and prior treatment history. More specifically, we sought to determine whether routinely collected medical records capture a clinically meaningful pattern consistent with emotional dysregulation as encountered in everyday psychiatric practice. The study was designed as a retrospective cross-sectional chart-review study with subgroup analysis within a single emergency inpatient cohort. This study should be considered an exploratory step preceding further research using standardized measures to assess the variables of interest more precisely.

## 2. Materials and Methods

### 2.1. Study Design and Data Sources

This was a retrospective cross-sectional chart-review study with subgroup analysis based on electronic medical records of patients aged 11–17 years who were hospitalized on an emergency basis at the Department of Child and Adolescent Psychiatry in Poznań between January and December 2024. The year 2024 was selected because it was the most recent complete calendar year available at the time of data extraction and allowed the inclusion of a full consecutive cohort of emergency psychiatric hospitalizations.

The study compared two clinically defined subgroups identified within this single emergency inpatient cohort. The reviewed records included admission interviews, psychiatric interviews, discharge summaries, psychological assessments, and information on previous psychiatric treatment.

Variables were coded on the basis of available clinical documentation. When a given symptom or experience was not documented, it was coded as absent. This approach reflects a common limitation of retrospective chart-review studies based on routine hospital records, as undocumented clinical features may not necessarily have been absent but, instead, may not have been recorded.

### 2.2. Participant Selection

In the first stage, two researchers reviewed the medical records of patients with the following ICD-10 diagnoses: F91 (conduct disorders), F92 (mixed conduct and emotional disorders), F93.9 (emotional disorder of childhood, unspecified), F60.9 (personality disorder, unspecified), F94.1 and F94.2 (attachment disorders), F32 (depressive episode), F33 (recurrent depressive disorder), F41.2 (mixed anxiety and depressive disorder), F43.1 (post-traumatic stress disorder), and F43.2 (adjustment disorders, including depressive reaction).

Adolescents with autism spectrum disorder, psychotic disorders, bipolar disorder, neurological disorders, intellectual disability, and mixed specific developmental disorders were excluded from the study. Primary eating disorders and active substance-related disorders were also excluded because they could substantially alter the affective, behavioral, and cognitive symptoms used in the symptom-based grouping procedure. However, adolescents were not excluded on the basis of past substance use alone if such use was not documented as an active disorder or clinically relevant problem during the index hospitalization.

If a patient underwent multiple hospitalizations during the study period, the patient was included in the analysis only once. The last hospitalization during the study period was selected as the index hospitalization because it was assumed to provide the most complete and clinically updated documentation while avoiding repeated inclusion of the same patient.

Any discrepancies regarding eligibility assessment and group assignment were resolved through discussion and consensus.

### 2.3. Definitions of the Study Groups

Emotional dysregulation was operationalized as a clinically defined, symptom-based profile characterized by the co-occurrence of the two core domains: affective dysregulation, reflected in affective instability and/or irritability, and behavioral dyscontrol, reflected in impulsivity and/or aggressive behavior.

This operationalization was informed by the literature conceptualizing emotional dysregulation as a transdiagnostic difficulty in modulating emotional responses, as well as by dimensional models emphasizing impaired behavioral control under emotional distress. Manifestations such as non-suicidal self-injury, suicidal ideation, suicidal behavior, dissociative symptoms, psychotic-like symptoms, and interpersonal difficulties were recorded separately. They were analyzed as additional indicators of clinical severity and complexity rather than as core defining features of the emotional dysregulation profile.

The comparison group consisted of adolescents with predominantly depressive presentations, documented in medical records, who did not meet criteria for the emotional dysregulation profile. Depressive presentations were identified on the basis of documented symptom patterns, broadly consistent with ICD-10 depressive symptomatology, which include at least two of the three core symptoms (depressed mood, anhedonia/loss of interest, and reduced energy or increased fatigability) and at least two additional symptoms documented in the medical records (such as low self-esteem, excessive guilt, pessimistic views of the future, impaired concentration, sleep disturbances, appetite disturbances, or suicidal thoughts and behaviors).

### 2.4. Group Assignment

After anonymizing the dataset, patients were assigned to one of two study groups:

1. The emotional dysregulation group, including patients who met criteria for both core domains of the emotional dysregulation profile.
2. The depressive presentations group, including patients with predominantly depressive presentations who did not meet criteria for the emotional dysregulation profile.

The grouping procedure was hierarchical, wherein patients who met criteria for the emotional dysregulation profile were assigned to the emotional dysregulation group even if they also presented with depressive symptoms. The depressive presentations group only included patients with predominantly depressive presentations who did not meet criteria for the emotional dysregulation profile.

Patients who did not meet the inclusion criteria for either group were excluded from the comparative analysis.

### 2.5. Clinical Variables and Adverse Childhood Experiences

In both groups, clinical variables were extracted from the medical records in order to more broadly characterize the symptom profiles. These variables included both symptoms used in the group assignment procedure and additional clinical characteristics not used for classification. The first category comprised the core features defining the emotional dysregulation profile and the depressive symptom pattern used to identify the comparison group. The second category included suicidal ideation, suicide attempts, non-suicidal self-injury, psychotic-like symptoms, feelings of emptiness, loneliness, anxiety symptoms, sleep disturbances, appetite disturbances, negative body image, memory problems, and interpersonal difficulties. Variables used for group assignment were presented to describe the clinical composition of the groups. However, because they contributed directly to the classification procedure, differences involving these variables were interpreted descriptively rather than as independent inferential findings.

Suicidal thoughts and behaviors were retained among the possible depressive symptoms because of their clinical relevance in adolescent depressive presentations. At the same time, suicidality was analyzed separately as a clinically important variable in the study population. Because suicidality could also contribute to the identification of depressive presentations, between-group comparisons involving suicidality should be interpreted with caution.

Medical records from both groups were also reviewed for the presence of adverse childhood experiences (ACEs), including traumatic events. The following ACE categories were taken into account: psychological, physical, and sexual abuse; exposure to domestic violence, including witnessing violence; neglect; parental separation; parental death; parental incarceration; parental alcohol abuse; parental mental illness; and peer violence.

ACE-related information was derived from routine clinical documentation, not from a standardized ACE assessment tool.

In the present study, childhood adversity was used as an umbrella term encompassing abuse, neglect, exposure to domestic violence, parental separation, and other ACE-related experiences.

### 2.6. Previous Psychiatric Treatment and Psychotherapeutic Care

Data regarding previous psychiatric treatment were collected for all included patients. The duration of prior treatment was categorized as follows: no previous treatment, less than 6 months, 6–12 months, 12–24 months, and more than 24 months. Information on previous psychiatric hospitalizations and the length of the index hospitalization was also collected. In addition, records were reviewed to determine whether the patient had received psychotherapeutic care before the index hospitalization.

### 2.7. Statistical Analysis

Statistical analyses were performed using PQStat Software version 1.8.2 (PQStat Software, Poznan, Poland). The distribution of continuous variables was assessed with the Shapiro–Wilk test. Comparisons between the two independent groups were conducted using the Mann–Whitney U test for non-normally distributed continuous variables and the chi-square test for categorical variables. When the assumptions for the chi-square test were not met, Fisher’s exact test was used. The significance level was set at α = 0.05 for all analyses.

Given the exploratory nature of the study, no formal correction for multiple comparisons was applied; thus, the findings should be interpreted with caution.

### 2.8. Ethical Considerations

According to the publicly available guidance of the Bioethics Committee at Poznań University of Medical Sciences, this retrospective non-interventional study based on existing medical records was exempt from formal ethics committee review, as it did not affect routine patient management.

## 3. Results

### 3.1. Baseline Demographic Characteristics

In 2024, there were 356 psychiatric hospitalizations at the Department of Child and Adolescent Psychiatry in Poznań. After applying the inclusion and exclusion criteria, consideration of repeated hospitalizations, and assignment to one of the two study groups, 139 adolescents were included in the final comparative analysis. In total, 85 were assigned to the emotional dysregulation group (61.2% of the final sample) and 54 (38.8%) to the depressive presentations group.

There were no significant differences between the emotional dysregulation and depressive presentations groups in terms of age or sex distribution: The median age was 13 years (Q1–Q3: 13–14) and 14 years (Q1–Q3: 12.25–14), respectively (Mann–Whitney U test, *p* = 0.708), and girls comprised 77.6% of the former group and 83.3% of the latter group (chi-square test, *p* = 0.415). The baseline demographic characteristics of the study groups are presented in Table 1.

### 3.2. Descriptive Symptom Profile of the Groups

To characterize the clinical composition of the groups, we first summarized the symptoms related to the classification procedure. These variables directly affected group assignment and should therefore be interpreted descriptively.

Consistent with the group definitions, affective instability and behavioral dyscontrol were markedly more frequent in the emotional dysregulation group. Affective instability was present in 83.5% of the adolescents in the emotional dysregulation group compared with 25.9% in the depressive presentations group; irritability was present in 85.9% versus 31.5%; physical aggression was noted in 76.5% versus 9.3%; and impulsivity was found in 77.6% versus 3.7%.

Depressive symptoms were more characteristic of the depressive presentations group, but they were also highly prevalent in the adolescents assigned to the emotional dysregulation group, pointing to substantial clinical overlap between the two profiles. Depressed mood was documented in all of the adolescents in the depressive presentations group and in 89.4% of the emotional dysregulation group, while anhedonia was present in 64.8% versus 36.5%, and reduced energy/increased fatigability was noted in 98.1% versus 49.4%, respectively.

Several associated depressive and neurovegetative symptoms were also more frequently documented in the depressive presentations group. For example, low self-esteem or feelings of inferiority were reported in 55.6% of the depressive presentations group compared with 34.1% of the emotional dysregulation group; pessimistic views of the future were presented by 16.7% versus 9.4%; feelings of guilt were noted in 16.7% versus 11.8%; impaired concentration was present in 48.1% versus 34.1%; sleep disturbances occurred in 83.3% versus 52.9%, including excessive sleepiness in 31.5% versus 7.1%; and appetite disturbances were present in 68.5% versus 44.7%, especially reduced appetite, which occurred in 51.9% versus 29.4%.

A detailed descriptive symptom profile related to group assignment is provided in Table S1.

### 3.3. Clinical Characteristics

Regarding additional clinical variables, several significant between-group differences were observed. Adolescents in the emotional dysregulation group more often presented with conflict-ridden relationships than those in the depressive presentations group (49.4% vs. 14.8%, *p* < 0.001). In contrast, social withdrawal was documented more frequently in the depressive presentations group than in the emotional dysregulation group (51.9% vs. 27.1%, *p* = 0.003). There were no significant differences between groups in terms of loneliness.

Regarding self-harm and suicidality, no significant between-group differences were found in the presence of suicidal ideation, which was documented in 90.6% of the adolescents in the emotional dysregulation group and 96.3% of those in the depressive presentations group (Fisher’s exact test, *p* = 0.316) or in the presence of suicide tendencies (80.0% vs. 85.2%, *p* = 0.438). However, adolescents in the emotional dysregulation group more often had a history of suicide attempts (72.9% vs. 50.0%, *p* = 0.006), including more frequent histories of more than two attempts (22.4% vs. 9.3%, *p* = 0.047). The number of suicide attempts was also higher in the emotional dysregulation group (median 1 [Q1–Q3: 0–2] vs. 0.5 [Q1–Q3: 0–1], Mann–Whitney U test, *p* = 0.012). Non-suicidal self-injury was common in both groups and did not differ significantly between them (81.2% in the emotional dysregulation group vs. 90.7% in the depressive presentations group, *p* = 0.125).

Among dissociative or psychotic-like symptoms, visual hallucinations were more frequent in the emotional dysregulation group (18.8% vs. 5.6%, *p* = 0.026). However, no significant between-group differences were found for other symptoms in this category, such as auditory hallucinations, derealization, ideas of reference, sense of being watched, or sense of presence.

No significant between-group differences were found for feelings of emptiness, anxiety symptoms, negative body image, or memory problems.

**Figure 1:**
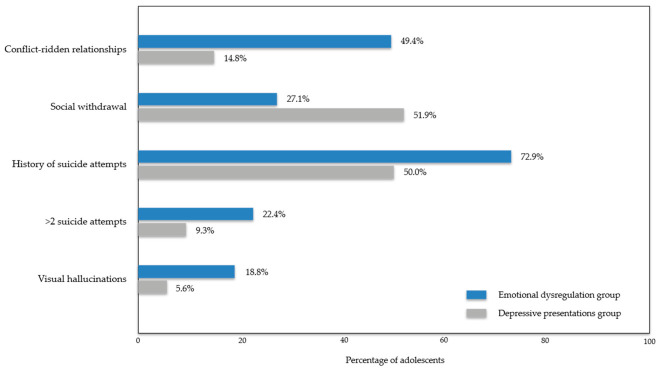
Selected between-group differences in clinical characteristics.

Additional detailed clinical characteristics beyond the core group-defining symptom profiles are presented in Table S2. Selected between-group differences in clinical characteristics are shown in Figure 1.

### 3.4. Adverse Childhood Experiences

Several indicators of childhood adversity were more frequently documented in the emotional dysregulation group.

When cumulative adversity indicators were analyzed, the emotional dysregulation group more often had a history of psychological abuse (40.0% vs. 20.4%, *p* = 0.016), physical abuse (37.6% vs. 18.5%, *p* = 0.017), and exposure to more than one type of violence (36.5% vs. 16.7%, *p* = 0.012). No significant between-group differences were found for sexual abuse or neglect.

Regarding family-related adversity, reports of violence toward the patient within the family were more common in the emotional dysregulation group than in the depressive presentations group (58.8% vs. 38.9%, *p* = 0.022). Adolescents in the emotional dysregulation group were also more likely to have witnessed domestic violence (30.6% vs. 14.8%, *p* = 0.035). Parental divorce or separation was more frequent in the emotional dysregulation group than in the depressive presentations group (65.9% vs. 42.6%, *p* = 0.007), as was a lack of contact with a living biological parent or parents (38.8% vs. 20.4%, *p* = 0.023). No significant between-group differences were found for parental death, parental alcohol use disorder, or parental mental illness.

No significant between-group differences were found for peer violence.

**Figure 2:**
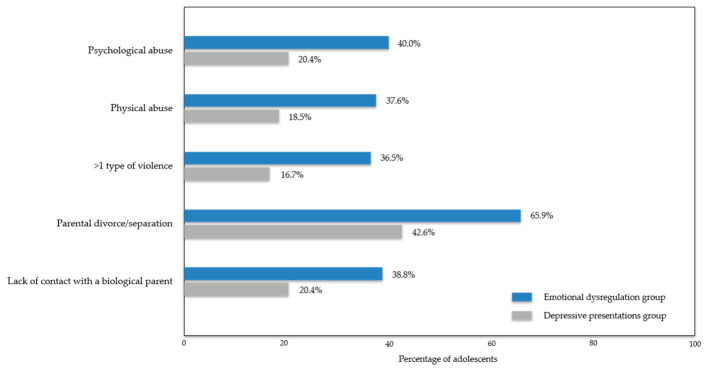
Selected between-group differences in adverse childhood experiences and family-related factors.

Detailed data on adverse childhood experiences and family-related adversity are shown in Table S3. Selected between-group differences in adverse childhood experiences and family-related factors are shown in Figure 2.

### 3.5. Previous Psychiatric Treatment and Psychological/Psychotherapeutic Care

Adolescents in the emotional dysregulation group had a more extensive history of psychiatric hospitalization. Although the median number of hospitalizations was 1 in both groups, the distribution differed significantly, indicating that adolescents in the emotional dysregulation group were more likely to have a history of repeated hospitalizations (Q1–Q3: 1–2 vs. 1–1, Mann–Whitney U test, *p* = 0.001). In contrast, the duration of the index hospitalization did not differ significantly between the groups (23 days [14–43] vs. 23 days [14.25–35.75], *p* = 0.532).

The timing of previous psychiatric treatment initiation also differed between groups. Adolescents in the depressive presentations group more often had initiated treatment within the previous 6 months (33.3% vs. 15.3%, *p* = 0.013), whereas adolescents in the emotional dysregulation group more often had been receiving treatment for more than 2 years (20.0% vs. 0.0%, *p* < 0.001). No significant differences were found in the intermediate treatment duration categories or in the proportion of previously untreated patients.

Contact with a psychologist or psychotherapist before the index hospitalization was common in both groups and did not differ significantly (87.1% in the emotional dysregulation group vs. 85.2% in the depressive presentations group, *p* = 0.754). Likewise, no significant between-group differences were observed for the specific form of pre-hospital psychological or psychotherapeutic care, including psychological care, school psychologist support, psychotherapy, or one-time consultations.

Detailed data on previous psychiatric treatment and psychological/psychotherapeutic care are presented in Table S4.

A summary of the key between-group differences across clinical characteristics, childhood adversity, and previous psychiatric treatment is presented in Table 2.

## 4. Discussion

Our findings suggest that, among adolescents hospitalized in an emergency psychiatric setting, it is possible to identify a clinically meaningful subgroup with a symptom profile suggestive of emotional dysregulation. Compared with adolescents in the depressive presentations group, this group appeared to have a more complex clinical picture. This complexity can be characterized by greater interpersonal difficulties, more severe suicidal behavior reflected in more frequent and more recurrent suicide attempts, more frequent exposure to several adversity-related experiences, and a more prolonged history of psychiatric treatment prior to hospitalization.

More specifically, adolescents in the emotional dysregulation group were more likely to present with conflict-ridden interpersonal relationships, whereas social withdrawal appeared to be more characteristic of the depressive presentations group. These findings are consistent with previous research linking emotion regulation difficulties to maladaptive interpersonal behaviors and interpersonal distress. Nevertheless, the exact nature of this association remains incompletely understood and is likely to be complex [28,29]. Schwartz-Mette et al. found concurrent and longitudinal associations between intrapersonal emotion regulation difficulties and maladaptive interpersonal regulatory behaviors, including excessive reassurance seeking, conversational self-focus, and negative feedback seeking [30]. Moreover, Adrian et al. showed that emotional dysregulation mediated the association between family and peer interpersonal difficulties and non-suicidal self-injury among psychiatrically hospitalized adolescent girls [31].

Importantly, suicidal ideation and non-suicidal self-injury were common in both groups. However, one of the most clinically relevant between-group differences was the more frequent history of suicide attempts in the emotional dysregulation group, including the greater likelihood of repeated attempts.

Esposito et al. found that, after controlling for mood disorder diagnosis, adolescents with a history of multiple suicide attempts differed from single attempters in terms of showing higher levels of anger, affect dysregulation, and serious self-mutilation [32]. Mittermeier et al. identified gender-specific pathways linking emotional dysregulation to suicidality in a community-based sample of German adolescents aged 11–14 years. In boys, emotional dysregulation was associated with a greater severity of suicidality indirectly via depressiveness, whereas in girls, it was linked to suicidality both directly and indirectly via depressiveness and non-suicidal self-injury [33].

Our findings may indicate that, in the subgroup identified in the present study, the clinical burden associated with emotional dysregulation is reflected less in the mere presence of self-harm-related phenomena and more in their severity and repetitiveness, as well as the broader interpersonal and behavioral context.

This interpretation is broadly consistent with previous findings from Poland and other clinical settings. For example, in a Polish study of adolescent psychiatric inpatients, Szmajda et al. found that non-suicidal self-injury, sexual abuse, and frequent interpersonal conflicts were associated with an increased risk of suicide attempts, which aligns with our observation that the adolescents in the emotional dysregulation group had a more frequent history of suicide attempts and a higher burden of adversity [34]. Likewise, in a U.S.-based sample of adolescent psychiatric inpatients, Hatkevich et al. reported that difficulties in emotion regulation were associated with both suicidal ideation and suicide attempts, supporting the view that emotional dysregulation may be associated not only with the presence of self-harm-related phenomena but also with their greater severity [35].

The present findings may also be interpreted in relation to previous studies on adolescents with borderline personality disorder (BPD) or borderline features in whom emotional dysregulation and self-harm are particularly prominent [36,37,38,39,40]. Prior research has suggested that adolescents with BPD may show levels of depression and hopelessness similar to those observed in individuals with major depressive disorder, while also demonstrating higher levels of anger, aggression, and impulsivity [40]. In addition, among depressed adolescents, the presence of borderline features has been associated with greater family difficulties, more frequent family interventions and hospitalizations, greater aggression, and poorer adaptation to peer groups [38]. Glenn et al. demonstrated that, among adolescent psychiatric inpatients with borderline features, affective instability was uniquely associated with suicidal ideation and attempts and differentiated ideators from attempters [41].

Although the present study was not designed to examine BPD as a diagnostic category, the available literature regarding this disorder may provide a clinically relevant point of comparison for understanding the broader and more complex profile observed in the emotional dysregulation group.

The more frequent documentation of psychological and physical abuse, domestic violence, parental separation, and lack of contact with a biological parent in the emotional dysregulation group may suggest an association between childhood adversity and a potentially more complex and chronic clinical profile. This interpretation is further supported by the longer duration of psychiatric treatment and the greater number of previous hospitalizations observed in this group.

Several mechanisms may contribute to the association between childhood adversity and emotional dysregulation. The previous literature suggests that early adversity may disrupt attachment formation and related caregiver–child processes, including emotion socialization and co-regulation, which are important for the development of emotion regulation and reflective capacities [42,43,44,45,46]. In this context, caregivers who are themselves a source of threat, violence, or neglect may be unable to provide the conditions necessary for the development of adaptive regulatory capacities. This interpretation is also consistent with findings showing that maltreated children may present greater emotion regulation difficulties, more aggressive behaviors, and more frequent peer rejection [47]. This perspective is further supported by Miu et al.’s (2022) meta-analysis, which found that emotion regulation difficulties partially mediate the relationship between childhood adversity and psychopathology [48]. The authors showed that ACEs were associated with greater overall difficulties in emotion regulation; more frequent use of maladaptive regulation strategies, particularly rumination and suppression; and less frequent use of adaptive strategies such as reappraisal. At the same time, they demonstrated a direct association between ACEs and psychopathology, suggesting that emotional dysregulation may represent just one of the mechanisms linking childhood adversity with later mental disorders [48].

Our findings are broadly consistent with the prior literature on childhood adversity and maltreatment. For example, Heleniak et al. demonstrated that emotional dysregulation may represent a transdiagnostic developmental pathway linking childhood maltreatment with both internalizing and externalizing psychopathology in adolescents [49]. Previous research also indicates that parental divorce is associated with adverse mental health outcomes in their children, including depression, anxiety, suicidal ideation and attempts, psychological distress, and substance use [50]. However, drawing causal conclusions is not possible given the retrospective, observational design of our study.

Our results suggest that routinely collected clinical records may capture a clinically meaningful pattern suggestive of emotional dysregulation, encompassing affective instability and behavioral dyscontrol. Although this subgroup overlaps clinically with adolescents presenting with depressive symptoms, its overall presentation appears broader and more complex. This observation may support the view that, in some acutely hospitalized adolescents, clinically relevant symptom constellations are not fully captured by ICD-10 categorical diagnoses and that routine discharge diagnoses may not always reflect the full complexity of the patient’s presentation.

From a clinical perspective, recognizing this pattern may have important practical implications. It may help clinicians to avoid conceptualizing such adolescents primarily in terms of depression alone, to broaden the differential diagnostic perspective, and to support more individualized treatment planning. Furthermore, it may encourage more careful assessment of the suicide risk, interpersonal functioning, and family context, all of which may be particularly relevant in adolescents presenting with emotional dysregulation.

Several limitations of our study should be acknowledged. Firstly, the study was retrospective and based on routinely collected medical records rather than standardized clinical assessments. Secondly, it included a relatively small sample from a single psychiatric ward for children and adolescents. As we did not perform stratified analyses by age group or sex, potential developmental and sex-related differences in the observed associations could not be assessed. In addition, the sample was predominantly female, which limits the generalizability of the findings, particularly to male adolescents. Future studies based on larger and more balanced samples should examine these issues in greater detail. Thirdly, no standardized tools were used to assess emotional dysregulation, associated clinical symptoms, or adverse childhood experiences. Furthermore, there is a potential bias in defining undocumented variables as absent. Finally, the exploratory nature of the analyses and the lack of correction for multiple comparisons require cautious interpretation of the findings.

Nevertheless, the study has several strengths. It was based on real-world clinical data and focused on an important and still insufficiently studied population of adolescents requiring emergency psychiatric hospitalization. It also attempted to identify a clinically meaningful transdiagnostic profile that may be highly relevant in routine practice but not easily captured within standard diagnostic categories.

## 5. Conclusions

Taken together, our findings suggest that adolescents presenting with features of emotional dysregulation may represent a clinically meaningful high-risk subgroup whose presentation differs from that of adolescents with predominantly depressive symptomatology. Compared with adolescents in the depressive presentations group, they appeared to show greater interpersonal difficulties, more recurrent suicide attempts, greater adversity burden, and a longer psychiatric treatment history, indicating a broader and more complex clinical profile.

Further prospective studies using standardized measures are needed to validate this profile and clarify its implications for diagnosis, risk assessment, and treatment planning.

## Figures and Tables

**Table 1:** Baseline demographic characteristics.

Variable	Emotional Dysregulation Group	Depressive Presentations Group	*p*
Final sample, *n* (%)	85 (61.2)	54 (38.8)	—
Age, years, median [Q1–Q3]	13 [13–14]	14 [12.25–14]	0.708
Female, *n* (%)	66 (77.6)	45 (83.3)	0.415

**Table 2:** Selected between-group differences in clinical characteristics, childhood adversity, and previous psychiatric treatment.

Variable	Emotional Dysregulation Group (*n* = 85)	Depressive Presentations Group (*n* = 54)	*p*
**Clinical characteristics**			
Conflict-ridden relationships	42 (49.4%)	8 (14.8%)	<0.001
Social withdrawal	23 (27.1%)	28 (51.9%)	0.003
History of suicide attempts	62 (72.9%)	27 (50.0%)	0.006
>2 suicide attempts	19 (22.4%)	5 (9.3%)	0.047
Number of suicide attempts, median [Q1–Q3]	1 [0–2]	0.5 [0–1]	0.012
Visual hallucinations	16 (18.8%)	3 (5.6%)	0.026
**Adverse childhood experiences/family-related adversity**			
Psychological abuse	34 (40.0%)	11 (20.4%)	0.016
Physical abuse	32 (37.6%)	10 (18.5%)	0.017
Exposure to >1 type of violence	31 (36.5%)	9 (16.7%)	0.012
Violence toward the patient within the family	50 (58.8%)	21 (38.9%)	0.022
Witnessing domestic violence	26 (30.6%)	8 (14.8%)	0.035
Parental divorce/separation	56 (65.9%)	23 (42.6%)	0.007
Lack of contact with biological parent(s)	33 (38.8%)	11 (20.4%)	0.023
**Previous psychiatric treatment**			
Number of hospitalizations, median [Q1–Q3]	1 [1–2]	1 [1–1]	0.001
Treatment initiated < 6 months before index hospitalization	13 (15.3%)	18 (33.3%)	0.013
Treatment duration > 24 months	17 (20.0%)	0 (0.0%)	<0.001

Data are presented as *n* (%) unless otherwise indicated. Quantitative variables are presented as median [Q1–Q3].

## Data Availability

Restrictions apply to the datasets (the data are part of an ongoing study).

## Supplementary Materials

The following supporting information can be downloaded at: https://www.mdpi.com/article/10.3390/jcm15093493/s1, Table S1: Descriptive symptom profile related to group assignment; Table S2: Clinical characteristics beyond the core group-defining symptom profiles; Table S3: Detailed adverse childhood experiences and family-related adversity; Table S4: Detailed previous psychiatric treatment and psychological/psychotherapeutic care.

## Abbreviations

The following abbreviations are used in this manuscript:

ACEs	Adverse childhood experiences
ADHD	Attention-Deficit/Hyperactivity Disorder
BPD	Borderline personality disorder
