# Supplementary material for: Clinical Characteristics of Adolescents Admitted to a Child and Adolescent Psychiatry Department in Poland: A Retrospective Chart Review
Source: J Clin Med. 2026 May 2;15(9):3493. doi: 10.3390/jcm15093493 (PMC13164166; doi:10.3390/jcm15093493)
Supplement: Supplementary file 1 [file jcm-15-03493-s001.zip › jcm-4251759-supplementary.pdf]

Supplementary Materials

Table S1. Descriptive symptom profile related to group assignment.

**Variable**	**Emotional dysregulation group (n = 85)**	**Depressive presentations group (n = 54)**
Affective instability	71 (83.5%)	14 (25.9%)
Irritability	73 (85.9%)	17 (31.5%)
Physical aggression	65 (76.5%)	5 (9.3%)
Impulsivity	66 (77.6%)	2 (3.7%)
Depressed mood	76 (89.4%)	54 (100.0%)
Anhedonia	31 (36.5%)	35 (64.8%)
Reduced energy/increased fatigability	42 (49.4%)	53 (98.1%)
Pessimistic views of the future	8 (9.4%)	9 (16.7%)
Feelings of guilt	10 (11.8%)	9 (16.7%)
Low self-esteem/inferiority	29 (34.1%)	30 (55.6%)
Concentration difficulties	29 (34.1%)	26 (48.1%)
Sleep disturbances	45 (52.9%)	45 (83.3%)
Excessive sleepiness	6 (7.1%)	17 (31.5%)
Difficulty falling asleep	33 (38.8%)	27 (50.0%)
Nocturnal awakenings	14 (16.5%)	12 (22.2%)
Appetite disturbances	38 (44.7%)	37 (68.5%)
Excessive appetite	4 (4.7%)	1 (1.9%)
Reduced appetite	25 (29.4%)	28 (51.9%)
Fluctuating appetite	9 (10.6%)	8 (14.8%)

Table S2. Clinical characteristics beyond the core group-defining symptom profiles.

**Variable**	**Emotional dysregulation group (n = 85)**	**Depressive presentations group (n = 54)**	**p**
Conflict-ridden relationships	42 (49.4%)	8 (14.8%)	<0.001
Social withdrawal	23 (27.1%)	28 (51.9%)	0.003
Loneliness	26 (30.6%)	21 (38.9%)	0.324
Self-harm thoughts and behaviors			
Suicidal ideation	77 (90.6%)	52 (96.3%)	0.316
Suicidal behavior	68 (80.0%)	46 (85.2%)	0.438
History of suicide attempts	62 (72.9%)	27 (50.0%)	0.006
>2 suicide attempts	19 (22.4%)	5 (9.3%)	0.047
Number of suicide attempts, median [Q1–Q3]	1 [0–2]	0.5 [0–1]	0.012
Non-suicidal self-injury	69 (81.2%)	49 (90.7%)	0.125
Dissociative / psychotic-like symptoms			
Visual hallucinations	16 (18.8%)	3 (5.6%)	0.026
Auditory hallucinations	25 (29.4%)	10 (18.5%)	0.149
Derealization	6 (7.0%)	1 (1.8%)	0.247
Ideas of reference	4 (4.7%)	2 (3.7%)	1.000
Sense of presence	1 (1.2%)	0 (0%)	1.000
Sense of being watched	7 (8.2%)	2 (3.7%)	0.482
Feelings of emptiness	28 (32.9%)	13 (24.1%)	0.272
Anxiety symptoms	68 (80.0%)	45 (83.3%)	0.619
Negative body image	28 (32.9%)	16 (29.6%)	0.685
Memory problems	12 (14.1%)	11 (20.4%)	0.336

Table S3. Detailed adverse childhood experiences and family-related adversity

Variable	Emotional dysregulation group (n = 85)	Depressive presentations group (n = 54)	p
Psychological abuse	34 (40.0%)	11 (20.4%)	0.016
Physical abuse	32 (37.6%)	10 (18.5%)	0.017
Sexual abuse	10 (11.8%)	5 (9.3%)	0.647
Neglect	13 (15.3%)	6 (11.1%)	0.487
Exposure to >1 type of violence	31 (36.5%)	9 (16.7%)	0.012
Violence toward the patient within the family	50 (58.8%)	21 (38.9%)	0.022
Witnessing domestic violence	26 (30.6%)	8 (14.8%)	0.035
Parental divorce/separation	56 (65.9%)	23 (42.6%)	0.007
Lack of contact with biological parent(s)	33 (38.8%)	11 (20.4%)	0.023
Parental death	6 (7.1%)	2 (3.7%)	0.410
Parental alcohol use disorder	10 (11.8%)	5 (9.3%)	0.647
Parental mental illness	22 (25.9%)	12 (22.2%)	0.624
Peer violence	17 (20.0%)	10 (18.5%)	0.824

Table S4. Detailed previous psychiatric treatment and psychological/psychotherapeutic care

Variable	Emotional dysregulation group (n = 85)	Depressive presentations group (n = 54)	p
Number of hospitalizations, median [Q1–Q3]	1 [1–2]	1 [1–1]	0.001
Length of index hospitalization, days	23 [14–43]	23 [14.25–35.75]	0.532
Treatment initiated < 6 months before index hospitalization	13 (15.3%)	18 (33.3%)	0.013
Treatment duration 6–12 months	12 (14.1%)	10 (18.5%)	0.488
Treatment duration 12–24 months	15 (17.6%)	4 (7.4%)	0.087
Treatment duration > 24 months	17 (20.0%)	0 (0.0%)	<0.001
No previous treatment	20 (23.5%)	20 (37.0%)	0.086
Treatment history unavailable	7 (8.2%)	2 (3.7%)	0.482
Previous contact with a psychologist /psychotherapist	74 (87.1%)	46 (85.2%)	0.754
Psychological care	30 (35.3%)	23 (42.6%)	0.388
School psychologist support	4 (4.7%)	4 (7.4%)	0.711
Psychotherapy	28 (32.9%)	10 (18.5%)	0.063
One-time consultation	10 (11.8%)	9 (16.7%)	0.412
